# Slipping while counting: gaze–gait interactions during perturbed walking under dual-task conditions

**DOI:** 10.1007/s00221-023-06560-6

**Published:** 2023-02-02

**Authors:** Carl Müller, Thomas Baumann, Wolfgang Einhäuser, Karl Kopiske

**Affiliations:** 1 Cognitive Systems Lab, Institute of Physics, Chemnitz University of Technology, Reichenhainer Str. 70, 09126 Chemnitz, Germany; 2 Physics of Cognition Group, Institute of Physics, Chemnitz University of Technology, 09126 Chemnitz, Germany

**Keywords:** Walking, Eye movements, Cognitive load, Perturbation, Dual task

## Abstract

Walking is a complex task. To prevent falls and injuries, gait needs to constantly adjust to the environment. This requires information from various sensory systems; in turn, moving through the environment continuously changes available sensory information. Visual information is available from a distance, and therefore most critical when negotiating difficult terrain. To effectively sample visual information, humans adjust their gaze to the terrain or—in laboratory settings—when facing motor perturbations. During activities of daily living, however, only a fraction of sensory and cognitive resources can be devoted to ensuring safe gait. How do humans deal with challenging walking conditions when they face high cognitive load? Young, healthy participants (*N* = 24) walked on a treadmill through a virtual, but naturalistic environment. Occasionally, their gait was experimentally perturbed, inducing slipping. We varied cognitive load by asking participants in some blocks to count backward in steps of seven; orthogonally, we varied whether visual cues indicated upcoming perturbations. We replicated earlier findings on how humans adjust their gaze and their gait rapidly and flexibly on various time scales: eye and head movements responded in a partially compensatory pattern and visual cues mostly affected eye movements. Interestingly, the cognitive task affected mainly head orientation. During the cognitive task, we found no clear signs of a less stable gait or of a cautious gait mode, but evidence that participants adapted their gait less to the perturbations than without secondary task. In sum, cognitive load affects head orientation and impairs the ability to adjust to gait perturbations.

**Supplementary Information:**

The online version contains supplementary material available at 10.1007/s00221-023-06560-6.

## Introduction

Locomotion, moving the body from one place to another, is one of the most fundamental forms of behavior (Fajen 2021). For humans, the most universal form of locomotion is walking. While universal, it is a complex task and depends on the constant perceptual exchange between information of the dynamic environment and the movement of the body (Gibson 1958). Thus, we continuously adjust our gait to the demands of our environment to move safely and efficiently through the world.

This way, most humans can traverse flat, uniform terrain, but also deal with slippery surfaces (Marigold and Patla 2002) or obstacles (Weerdesteyn et al. 2004). To achieve this, they make use of many different sources of information, most prominently visual cues (Laurent and Thomson 1988; Patla 1997). Especially in difficult terrain, this sensory information is helpful because it is usually available at a distance, providing important information about potential threats to stability early on (Fajen and Warren 2003; Gibson 1958) and so enabling preemptive gait adjustments (Warren et al. 1986) to prevent potential damage. Such obstacles or sudden hazards humans have to respond to are in experimental environments often simulated by induced motor perturbations (Kopiske et al. 2021). For example, participants can be made to slip or stumble to increase the difficulty and complexity of the experimental situations. This enables us to investigate walking and sampling of information—such as through gaze adjustments—in difficult conditions, while maintaining high experimental control and participants’ safety.

Processing all these sensory inputs simultaneously (e.g., visual as well as haptic cues in difficult terrain) on the one hand facilitates walking, but it also requires cognitive resources (Hausdorff et al. 2005). In advanced age, even ordinary walking and the required real-time adaptation can be a complex task that requires higher-level cognitive input (Hausdorff et al. 2005). But what happens if we have to manage other daily actions while walking? Numerous actions from simple talking (Hyndman 2004) to looking at a mobile phone (Ioannidou et al. 2017) distract from walking because cognitive resources are used elsewhere. In fact, a large proportion of everyday tasks consist of precisely this simultaneous execution of cognitive and motor tasks such as walking (Hunter et al. 2018). So as walking becomes more difficult when combined with cognitive tasks, an important question arises: what happens if not enough cognitive resources are available?

A lot of research on motor control, walking itself, and falling (Hausdorff et al. 2005) has focused on how cognition and walking interact. One approach combining these two is through dual-task paradigms, which consist of the simultaneous execution of a cognitive secondary task while walking to study their interaction (Montero-Odasso et al. 2012a, b). If the cognitive load exceeds the participant’s cognitive capacity, either the performance of the primary task (motor task), the secondary task (cognitive task) or both is reduced (Yogev-Seligmann et al. 2008). For example, clinical walking tests using dual-task paradigms have found a strong impact on gait changes (Hyndman 2004), decreasing gait stability and thus increasing the risk of falling especially in older adults (Kressig et al. 2008). Gait instability indeed is one of the most common factors of fall risk for hospital falls (Oliver 2004). There are many approaches for dual-task paradigms on walking and the extent to which performance is reduced depends on the type and difficulty of the cognitive task. Besides influences of auditory tasks (Beurskens et al. 2016) and verbal fluency tasks (Bahureksa et al. 2017; Montero-Odasso et al. 2012a, b), a variety of different arithmetic tasks (Hunter et al. 2018; Montero-Odasso et al. 2012a, b; Springer et al. 2006) have often been used. In addition to the task itself, it is important to choose the right level of difficulty where secondary tasks are not too easy, but still doable. Bahureksa et al. (2017) detected more pronounced effects of a decreasing gait velocity for serial subtraction in steps of seven compared with steps of one while investigating the difference between mildly cognitively impaired and cognitively unimpaired participants.

Cognitive tasks do not across-the-board increase fall risk but often lead to a shift to a more cautious gait mode. For example, Soangra and Lockhart (2017) found increased double-support time (time during which both feet are on the ground) when participants completed an additional backward-counting task. Similarly, arithmetic tasks like serial subtraction in steps of seven reduced gait velocity (Hunter et al. 2018; Montero-Odasso et al. 2012a, b; Springer et al. 2006) or resulted in an increased gait variability (Montero-Odasso et al. 2012a, b). In sum, previous findings suggest that under certain conditions, cognitive dual tasks increase the effect of gait perturbations due to the cognitive distraction, therefore increasing the risk of falling. Under other conditions, they may lead participants to walk more cautiously thereby making them less susceptible to perturbations. This raises the question when the increased caution outweighs the increased risk.

A typical response to an increased *walking* difficulty is sampling *more* visual information—looking more closely where to take the next steps. This has been shown for terrain negotiation, where rougher terrain resulted in lowered gaze on average (Matthis et al. 2018; 't Hart and Einhäuser 2012), as well as for experimentally perturbed walking (Kopiske et al. 2021), where perturbations not only evoked immediate gaze responses, but also led participants to look down more on average between slips, especially when perturbations were not visually cued ahead of time. When a visual cue to perturbation was provided, participants tended to fixate it, up until shortly before the perturbation. A typical response to performing challenging *cognitive* tasks on the other hand is to *reduce* sensory (and especially visual) input. As Glenberg et al. (1998) showed, participants completing memory and arithmetic tasks tended to gaze toward the ceiling during the more difficult moments of the task, away from the stimuli. This raises the question: in the common scenario of walking under increased difficulty, and while also completing a cognitive task, do participants reduce or maximize the sampling of visual information?

In the present study, we aimed at a scenario that resembles natural walking as closely as possible, while ensuring participant safety and maintaining experimental control and reproducibility. So, we combined a paradigm in which slipping is induced through perturbations upon foot placement (Kopiske et al. 2021) with a cognitive task. Using this paradigm, we tested whether (i) increasing cognitive load leads to more pronounced gaze responses to perturbation/slipping events, and (ii) whether increasing cognitive load leads either to a greater impact of perturbations on dynamic stability or to participants adopting a more cautious gait mode to compensate such potential effects. To this end, we examined the impact of a cognitive task (serial subtraction in steps of seven) on gait stability and gaze orientation while walking, while the predictability of perturbations was manipulated through visual cues (transparent blueish rectangles on the virtual road). To do this, we asked participants to walk on a dual-belt treadmill (while secured by a safety harness) through a virtual environment. The setting was chosen to be as naturalistic as possible, while still allowing us to induce slipping under full experimental control and without imposing any safety hazards on the participants. It included a dynamic environment with a path, lateral walls, grass and sky as well as a floor projection to provide a naturalistic experience. Meanwhile, their gait was repeatedly perturbed to induce slipping (using a procedure established previously by Kopiske et al. 2021). We assessed the relevant gaze and gait parameters at three different time scales: (a) immediately in a 3-s time window after each perturbation, (b) over the entire 5-min blocks excluding the 3-s time windows, and (c) how the parameters in these 3-s time windows varied over the 5-min block for adaptive changes to the perturbation and between 5-min blocks of the same type. On each time scale, we analyzed eye, head, and body movements to look at persistent changes. Previously, we had shown that participants respond to such perturbations by adapting their gaze both directly and long-term, and differently depending on whether there were visual cues to give advance notice of the perturbation or not (Kopiske et al. 2021). Specifically, immediate changes showed a lowered gaze while perturbations occurred, mainly driven by changes in head orientation. Longer-term, the presence of a visual cue resulted in a raised gaze over the 5-min blocks.

If the adverse effects of increased cognitive load are not offset by a more cautious gait mode, one would predict a stronger reaction of gaze and gait parameters to the perturbation while performing a cognitive task than without secondary task. Alternatively, participants might switch to such a cautious gait mode and display less pronounced slip responses. As we investigated young and healthy participants, we expected the cognitive task to be performed virtually error free, while inducing an appropriate level of cognitive distraction. We also expected an increased variability of the perturbation responses during the dual-task conditions, as has been shown for dynamic gait stability (Montero-Odasso et al. 2012a, b). This expectation applies to between-subject variability, as individual differences in two tasks rather than in one may contribute to inter-individual differences, as well as to within-subject variability as available resources may fluctuate between the two tasks over time, increasing the measured variability on either.

## Methods

### Participants

Participants were recruited via a TU-Chemnitz online mailing list and could participate if they had self-reported normal or corrected-to-normal vision (≤ ± 7 dpt when uncorrected, contact lenses were permitted), no neurological or walking impairments, and a body mass of 130 kg or less. Visual and body mass-based exclusion criteria were based on the device limits of the eye tracker and the treadmill, respectively. All participants reported being sufficiently rested and focused in a questionnaire prior to the experiment, were naïve to the hypotheses and debriefed after the experiment. We aimed for a power of 80% (Cohen 1988) which, given $\alpha =.05$ and Cohen’s $f=0.25$ (a realistic estimate based on previous work, Kopiske et al. 2021), required a sample size of $N=24$. A total of 27 participated, as after inspecting data quality, but prior to any hypothesis-related analysis, data of three participants had to be excluded due to a high proportion of missing eye-tracking data (> 20% missing values, same cutoff as used in Kopiske et al. 2021).

The analyzed sample of *N* = 24 included 14 women and 10 men with an average age of 24.3 years (between 19 and 34), average height 173 cm ± 9 cm (standard deviation), average body mass 68 kg ± 15 kg and average leg length 94 cm ± 6 cm. These biometric measurements were required for modeling motion tracking. For participation, participants received either course credit or a monetary reimbursement of 8€/h. All experimental procedures were approved by the Chemnitz University of Technology, Faculty of Behavioral and Social Sciences ethics committee (case no.: V-314-PHKP-WETGRAIL01-17012019). Participant data were protected following the guidelines for data management and data sharing of the German DGPs (Gollwitzer et al. 2020).

### Environmental setup and materials

**Fig. 1:**
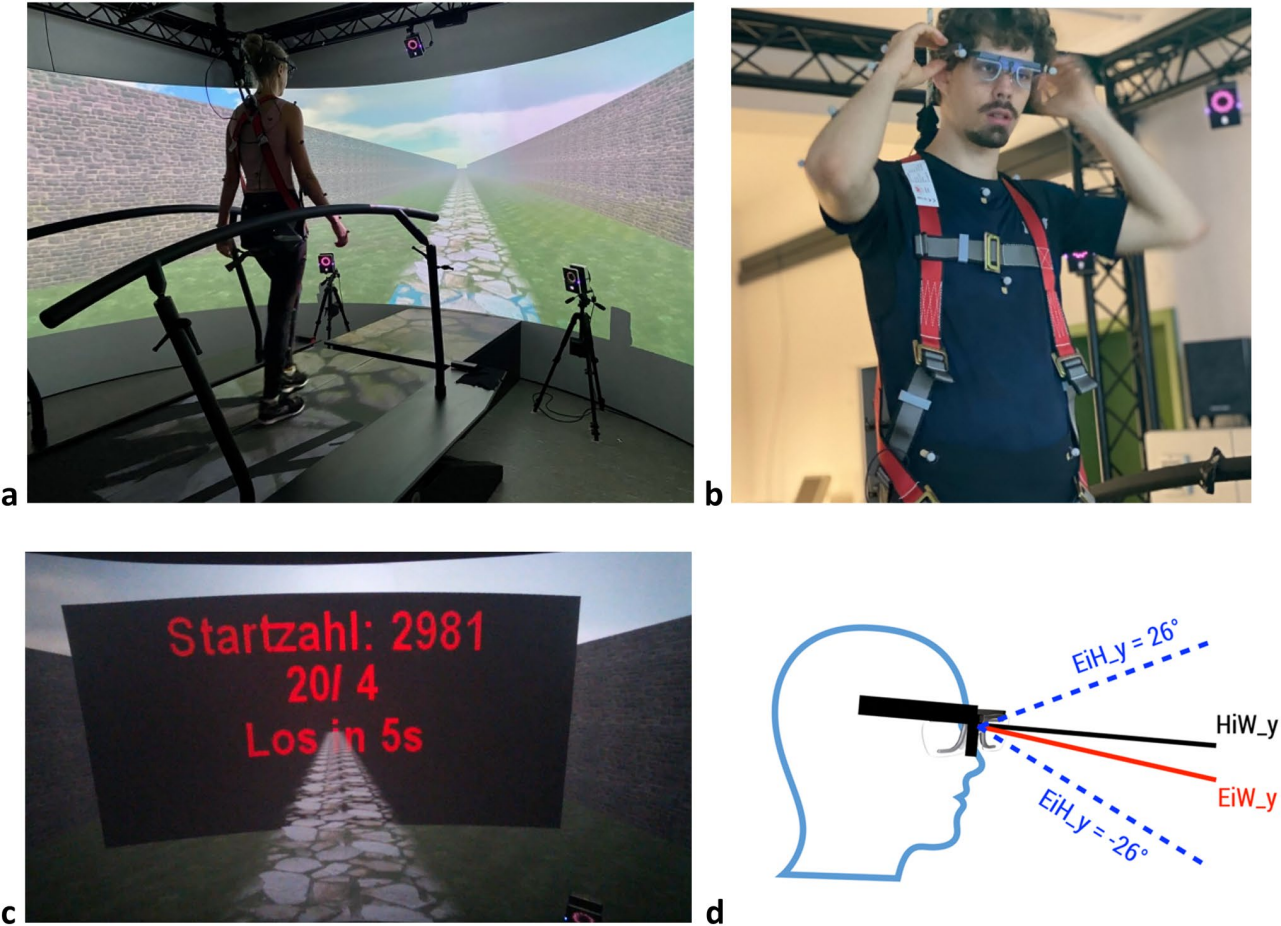
Virtual environment, marker positions, and data obtained. **a** Participant walking on the treadmill along the endless road, secured with a harness to prevent potential falls. A transparent blue square (seen here on the transition between the treadmill and the screen) simulated an ice plate which cued split-belt perturbations for the leg-side participants first stepped in it with. Infrared cameras around the treadmill recorded the three-dimensional positions of the markers. **b** Front-view of a participant, showing the mobile eye tracker and the positions of the passive markers, attached to the eye tracker and relevant body segments for motion capture. **c** The countdown indicates the time to starting the treadmill. Conditions in which participants were instructed to perform the cognitive task (“c1”), were indicated by the presence of a starting number above the countdown, and to start counting backward at the displayed number. Example shows a c1 condition, the starting number displayed on top (“Startzahl”, German for “starting number”). **d** Motion-capture data from the four markers on the eye-tracking glasses was used to calculate head orientation (and its vertical component HiW_y) and the gaze vector (with vertical component EiH_y) in degrees, as well as the position of the cyclopean eye. Combining HiW and EiH provides the gaze orientation in the real world EiW (and its vertical component EiW_y)

The experiment was conducted in a GRAIL (Gait Realtime Analysis Interactive Lab; Motek Medical, Amsterdam, Netherlands) gait laboratory at TU Chemnitz for high-precision real-time motion measurement. The GRAIL combines a dual-belt treadmill with a virtual 240° projection screen to simulate an environment for walking (Fig. 1a). Each belt could be accelerated independently at 15 m/s^2^. When accelerating or decelerating the belt to induce or end a motor perturbation, acceleration was constant (i.e., velocity changed linearly) at this maximum. From the signal to start an acceleration, it takes approximately 50 ms for the belt to actually commence the acceleration, with a variation of less than the precision of sampling. (i.e., latency varies less than 4 ms across trials). This latency was measured independently by us with the motion-capture device and corresponds to data found in the literature (Sessoms et al. 2014). Ground-reaction forces were measured at 250 Hz using force plates below the belts. These forces were used to trigger perturbations, using a threshold of 100 N. The visual environment was a simple endless road with lateral walls, which was projected on a curved screen at a distance of 2.5 m from the center of the treadmill at 60 Hz, as well as being visible on the treadmill via floor projections. The virtual horizon was at a height of 1.25 m.

For motion capture, 39 retro-reflective markers were placed on participant`s body segments according to the Vicon Plug-In Gait full-body model (Vicon Motion Systems, Yarnton, UK) (Fig. 1b). We placed the markers directly on participant`s skin or tight-fitting sportswear, and they were always applied by the same person to increase reliability (McGinley et al. 2009). Ten infrared cameras placed on different positions around the treadmill recorded the exact three-dimensional positions of the markers at a rate of 250 Hz. Head orientation was captured using four markers attached to a Tobii Pro Glasses 2 mobile eye tracker (Tobii Pro AB, Stockholm, Sweden), which recorded eye position. The Tobii Pro Glasses 2 allow accurate eye tracking at 100 Hz with a large field of view (82° horizontal, 52° vertical) and an accuracy of 0.73° at 3 m distance according to the manufacturer. Calibration was done using a standard calibration card and validated before and after each block using a grid of 20 fixation points on the screen. This validation procedure was used to apply a drift-correction to the recorded eye positions (supplementary movie S1), described in more detail in the paragraph “Data processing and variables”.

### Procedure

For each participant, first we took biometric measurements including height and leg length and applied markers. Following a standard calibration procedure (consisting of a T-pose and ca. 5 s of walking), the motion-capture model was calibrated. At the start of the experiment and after each break, the eye tracker was (re-)calibrated and prior as well as after each block the validation procedure was conducted.

Participants first performed a baseline block of 150 s (2 min 30 s) of unperturbed walking, followed by eight experimental blocks of 5 min of perturbed walking. In the end, again a baseline block had to be completed. Walking started with an acceleration of the treadmill to base speed of 1 m/s in 5 steps of 0.2 m/s, following a countdown. Participants wore a safety harness connected to a ceiling hook throughout walking blocks.

In a 2 × 2 design, we manipulated independently whether participants would be given visual cues to perturbations or not (factor *visual cue*, denoted as “v1” and “v0”, respectively) and whether they had to complete a cognitive task, counting backward loudly in steps of seven from a random starting number between 2000 and 3000 (factor *cognitive task*, denoted as “c1” and “c0”, respectively). Numbers were chosen so that participants would not be able to reach three-digit numbers within the 5 min provided. Participants were free to emphasize speed or accuracy in counting as they preferred. In blocks with a cognitive task, a starting number was displayed above the countdown prior to the start of walking (Fig. 1c). The visual conditions correspond to those of Kopiske et al. (2021), and are here crossed with the cognitive task manipulation, which had not been used earlier. Each of the four resulting conditions was presented twice for eight experimental blocks, with the first four experimental blocks always containing each condition once and the order being counterbalanced across participants (each of 4! = 24 possible permutations presented to one participant). The last four experimental blocks also contained each condition once, always in reverse order of the first four blocks.

In each experimental block, motor perturbation occurred between 12 and 20 m walking distance apart, 16 m on average. In half the blocks, perturbations were visually cued by transparent blue 1-m × 1-m squares on the road (supplementary movie S2). These perturbations consisted of accelerating one belt from the baseline speed of 1 m/s to 2 m/s at a constant acceleration of 15 m/s^2^ for a single step.^1^ That is, the motor perturbation was triggered and started immediately when participants stepped into a square—visible as blue “ice” plate in “v1” conditions (Fig. 1b), invisible in “v0” conditions—for the corresponding leg side they first stepped in with and ended with the belt being decelerated to baseline speed once the perturbed leg was lifted off the belt.

### Data processing and variables

In 2 of the 24 participants, one block each had to be excluded from analysis, as the participant’s hair had slipped over the markers attached to the mobile eye tracker. In the remaining data, the median proportion of missing eye data (which included blinks) was 10.8% during unperturbed walking and 13.0% in the reported 8-s windows around slips. We applied a cubic-spline interpolation and a Savitzky–Golay Filter (Savitzky and Golay 1964) with a window of 110 ms to smooth the signal. The same procedure was applied to the kinematic data, where all relevant markers at the head, foot, and pelvis had < 0.1% missing data (maximum for any block: 9.3%).

Data from the validation procedure (extracted from the headcam video) showed a median absolute deviation of the gaze position from the positions of the calibration spheres of 1.05°, with no signs of drift (−0.07° per block) and no substantial bias for either the median vertical error (+ 0.21°, with the maximum absolute value of any block being 3.6°) or the median horizontal error (+ 0.31°, maximum absolute value of 7.6°). We applied the corresponding correction to the eye-position data on a block-wise basis. We also used the headcam video to detect for each participant the angle between the back and front markers on the eye tracker when the head was not inclined (which differed slightly depending on the fit of the glasses to the head and the exact position of the markers, as the Tobii glasses’ sidepieces are not horizontal or perfectly straight) and aligned the data accordingly.

We used the vertical component of (i) head orientation (“head-in-world”, HiW_y), (ii) eye position (“eye-in-head”, EiH_y), and (iii) gaze in allocentric coordinates (“eye-in-world”, EiW_y) in degrees as our main variables. These variables were computed the same way as in Kopiske et al. (2021), as depicted in Fig. 1d: HiW_y was defined as the mean slope of the two vectors between the back-head and the front-head markers attached to the mobile eye tracker, with HiW_x being the angle between the vectors from the markers on the right side of the glasses to those on the left and *x*-axis of the coordinate system. The gaze vector, relative to the field of view of the eye tracker was assumed to originate from a cyclopean eye calculated as the mean position of the two front markers. Combining HiW and EiH provides the gaze orientation in the real world EiW (and its vertical component EiW_y).

For gait stability, based on the model of a double inverted pendulum (Mochon and McMahon 1980), we computed the (iv) anterior–posterior margin of support (MOS_ap_). This is calculated as the minimum distance between the anterior or posterior foot marker when first touching the ground (base of support) and the center of mass (CoM, mean position of the hip markers). The CoM was then adjusted for its movement and the angular frequency of the pendulum (Hof et al. 2005; Whittle 1997) and derived from the heel–pelvis distance $l$ and gravity constant $g$. The adjusted center of mass (XCoM) was calculated as

1 $$\mathrm{XCoM}=\mathrm{CoM}+\frac{\dot{\mathrm{CoM}}}{\sqrt{\frac{g}{l}} }.$$

The experimenter noted errors in counting, for which he was aided by a display of the correct numbers on the control display (unavailable to the participant). To further analyze counting rate, we bandpass-filtered the sound signal of our recordings at 150–1500 Hz to preserve speech but remove treadmill noises, and then used the function speechDetect from the MATLAB (Mathworks Inc., Natick, MA, USA) audio toolbox (Giannakopoulos et al. 2009) to detect the onsets and offsets of the participant speaking.

We analyzed the effects of visual cue and cognitive task on each of our main parameters with a 2 × 2 repeated-measures analysis of variance (rmANOVA). These were conducted separately for 8-s windows around slips (5 s prior and 3 s after each perturbation), using peak-trough differences within these windows, and for parameter means during unperturbed walking in the remaining time windows (excluding the 8-s slip window) between slips.

## Results

Participants walked through an endless road scene with moderate speed (1 m/s) in a virtual environment, dealing with quasi-randomly occurring motor perturbations which were either visually cued or not (factor *visual cue*). In addition, participants were instructed to count backward in steps of seven (counting units) in half of the blocks as a cognitive secondary task (factor *cognitive task*). We consider the effect of perturbations on gaze and gait on three different time scales: immediate (event-based) adjustment to the perturbation, within-block adaptation to the perturbation and long-term (across-block) adaptation.

### Event-related gaze and gait patterns around slips

#### Gait

**Fig. 2:**
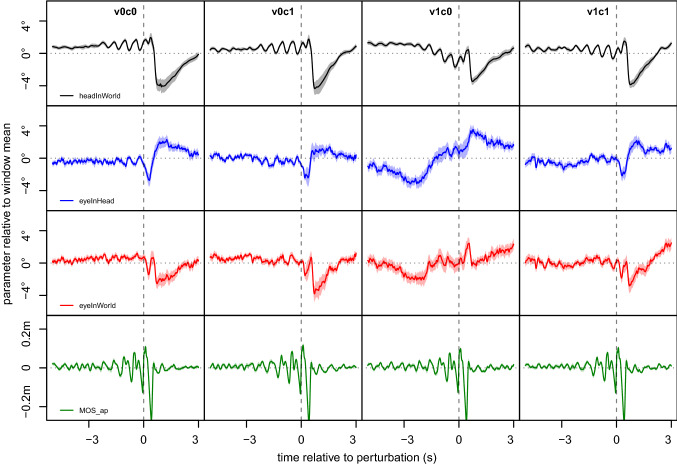
Average gaze and gait parameters relative to slips. Trajectories of the relevant parameters HiW (top row), EiH (second row), EiW (third row) and MOS_ap_ (bottom row) in an 8-s time window around the perturbations for vertical orientation, ordered by condition, given at the top of each column. The shaded areas indicate the standard error of the mean (SEM) across all participants, the *x*-axis the time relative to the perturbation (dashed vertical lines), and the *y*-axis shows the parameter over time relative to the window mean. Slip responses to perturbations for HiW and EiH were strong but partially compensatory, also reflected in EiW. Gait stability decreased after perturbations showed in MOS_ap_, confirmed that these induced slipping. Note that the SEM is so small for MOS_ap_ that shading may only be visible when zooming in the high-resolution version of this figure

For immediate effects of the perturbation, we analyzed the peak-trough differences of our main variables in fixed 8-s time windows (between 5 s prior and 3 s after, as in Kopiske et al. 2021) to provide measures of how strongly a parameter varied during that time. As expected, we found that the induced motor perturbations reliably triggered slipping, confirmed by the time course of the MOS_ap_ (Fig. 2, bottom row) with the typical oscillatory pattern of steps before perturbations, reduced stability of gait associated with more variability around slips, but then rapid gait stabilization again.

MOS_ap_ was neither affected by *visual cues* (*F*(1, 23) = 0.22, *p* = 0.641) nor by the *cognitive task* (*F*(1, 23) = 2.46, *p* = 0.131) nor was there an interaction between the factors (*F*(1, 23) = 2.55, *p* = 0.124). This implies that there is no evidence for a difference in motor patterns of slipping irrespective of participants being cued or cognitively distracted. The time course shows that the slip consistently occurred within 200 ms after the perturbation (the time of perturbation corresponding to *t* = 0 in the event-based analyses).

#### Head-in-world

We tested whether having to complete a cognitive task would influence patterns of head responses to motor perturbations. Indeed, these were less pronounced while counting, as shown by the main effect of *cognitive task* (*F*(1, 23) = 4.48, *p* = 0.045) on the peak-trough differences. Conversely, we see no significant effect of the *visual cue* (*F*(1, 23) = 1.13, *p* = 0.299), that is, no evidence of tracking of visual cues through head movements. We see no *visual cue* × *cognitive task* interaction (*F*(1, 23) = 1.29, *p* = 0.269), although descriptively the trajectories of the v1c0-conditions looks slightly different (Fig. 2).

#### Eye-in-head

**Fig. 3:**
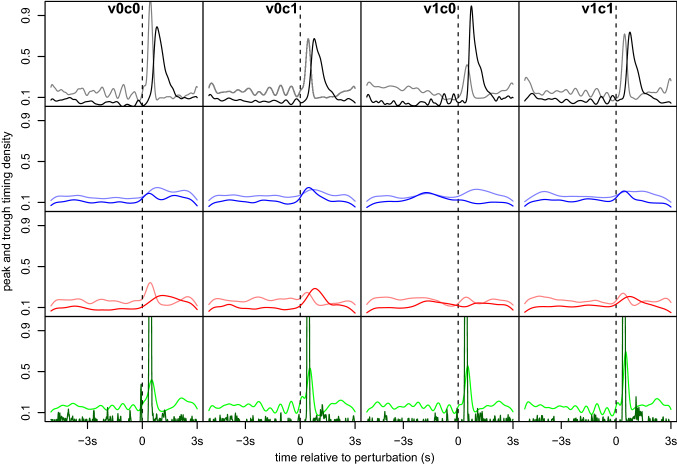
Distribution densities of peak and trough timing. We determined the respective time points of the peak and the trough of each slip and calculated the densities, with bandwidths chosen using Sheather and Jones’ (1991) method. Dark lines show densities for the trough, lighter lines for the peak. We see a much more concentrated distribution for motor measures HiW_y (black) and MOS_ap_ (green), with peak and trough in quick succession after the perturbation. For EiH_y (blue) and EiW_y (red) the distributions are much more spread out, although here too, peaks and troughs tend to occur after the perturbation, with the notable exception of EiH_y, condition v1c0, where the trough occurs predominantly before the perturbation.

Looking at the eye movements by using mobile eye tracking, the *visual cue* affects eye movements (*F*(1, 23) = 12.58, *p* = 0.002), another indication of visual tracking, but the *cognitive task* did not (*F*(1, 23) = 0.44, *p* = 0.513) with no significant interaction between the factors (*F*(1, 23) = 1.73, *p* = 0.202). Vertical eye position shows a clear downward shift after the perturbation in all conditions (Fig. 2, second row), except when the presence of a visual cue was combined with the absence of the cognitive task (condition v1c0). Here unlike all other conditions, the downward shift occurred markedly prior the perturbation, see Fig. 3. However, repeating the peak–trough analyses using only the 3 s after each perturbation showed no evidence of a clearly stronger or weaker downward shift depending on the condition, with no main effect for visual cue, *F*(1, 23) = 1.66, *p* = 0.211, or cognitive task, *F*(1, 23) < 0.01, *p* = 0.965, or an interaction, *F*(1, 23) = 0.12, *p* = 0.738.

#### Eye-in-world

The gaze as a combination of the previously described head and eye movements displayed a smaller reaction to the perturbation with *visual cues* (*F*(1, 23) = 6.02, *p* = 0.022) than without them. This was independent of the presence or absence of a *cognitive task* (*F*(1, 23) = 0.07, *p* = 0.798; interaction: *F*(1, 23) = 0.59, *p* = 0.452). Descriptively, the event-related patterns for EiW are mainly affected by HiW (Fig. 2) but with a less pronounced downward shift, as EiH shows a partially compensatory pattern to perturbations.

#### Gaze and gait

As in Kopiske et al. (2021), we tested whether a less stable gait and a more variable gaze are more likely to occur together. Mean correlations over conditions of the peak-trough differences for gaze and gait between participants were low, as they had been in Kopiske et al. (2021). For *r*_MOS,HiW_ = 0.18 (−0.08, 0.42) as well as *r*_MOS,EiW_ = 0.10 (−0.19, 0.37) even lower correlations were found, which replicates (Kopiske et al. 2021) the finding that perturbations that destabilize gait more effectively do not necessarily have a stronger effect on gaze parameters than less effective perturbations.

#### Cognitive dual task

**Fig. 4:**
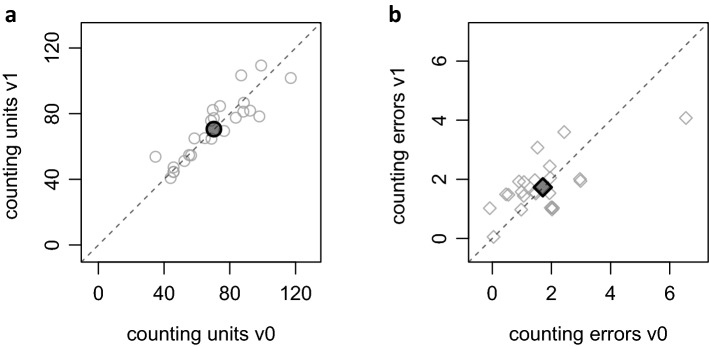
Mean counting units and errors by visual condition. Counting units (**a**) and counting errors (**b**) of the cognitive task, during 5-min perturbed walking. Each small dot represents one participant. Blocks with visual cue (v0c1) are on the *x*-axis, blocks without visual cue (v1c1) on the *y*-axis give the *x*-coordinates. Thus, points on the diagonal indicate perfectly equal performance in v0 and v1 blocks, while points above the diagonal indicate faster (**a**) or more error-prone (**b**) counting when visual cues were given. Large, filled dots show overall means. These are almost perfectly on the diagonal, suggesting that participants on average did the counting task equally well and equally fast in v0 and v1 blocks, respectively.

Descriptive analyses of the dual task provided mean counting errors per block of 1.7 (SD 1.3) with average 70.4 (SD 21.4) units counted per 5-min block, consistent with typical findings of about 14 counts per minute while counting backward (Holding 1989). Variability in counting units and counting errors was much higher between participants (SD = 1.1) than between conditions (SD = 0.8; see also Fig. 4). Moreover, the high accuracy indicates that participants were sufficiently focused on the cognitive task. Approaching perturbations showed no clear effect on counting, as gap times between syllables only marginally decreased when participants saw a visual cue approaching with a median gap time of 480 ms in the 5 s before a slip when a visual cue was given, compared to 495 ms in blocks with visual cue (v1) and 547 ms during blocks without (v0). The difference between the two conditions was not statistically significant, *t*(23) = 0.05, *p* = 0.959.

### Mean gaze and gait parameter per block

Gaze and gait parameters showed immediate event-based effects to perturbations, depending either on visual cues or the cognitive task, in fixed time windows around the slips. Next, we tested whether there are also longer-term effects of the mean parameters over entire blocks, while excluding the slip time windows.

#### Gait

Gait stability (MOS_ap_) showed no significant effect of the *cognitive task* (*F*(1, 23) = 3.11, *p* = 0.091). Also neither the *visual cue* (*F*(1, 23) = 0.03, *p* = 0.874) nor the interaction (*F*(1, 23) = 3.01, *p* = 0.096) showed a significant impact on the MOS_ap_. Descriptively, gait stability was somewhat higher in blocks with a *cognitive task* (v0c1: 0.049 m; v0c0: 0.038 m) looking at the absolute means per block (Table 1).

**Table 1:** Absolute means per condition for each block with the excluded 8-s time window for HiW, EiH, EiW and MOS_ap_.

	Mean per block			
	HiW	EiH	EiW	MOS_ap_
v0c0	−6.7°	6.1°	−0.5°	0.038 m
v0c1	−4.1°	7.9°	3.8°	0.049 m
v1c0	−6.7°	5.8°	−0.9°	0.044 m
v1c1	−4.3°	7.4°	3.1°	0.044 m

#### Head-in-world

**Fig. 5:**
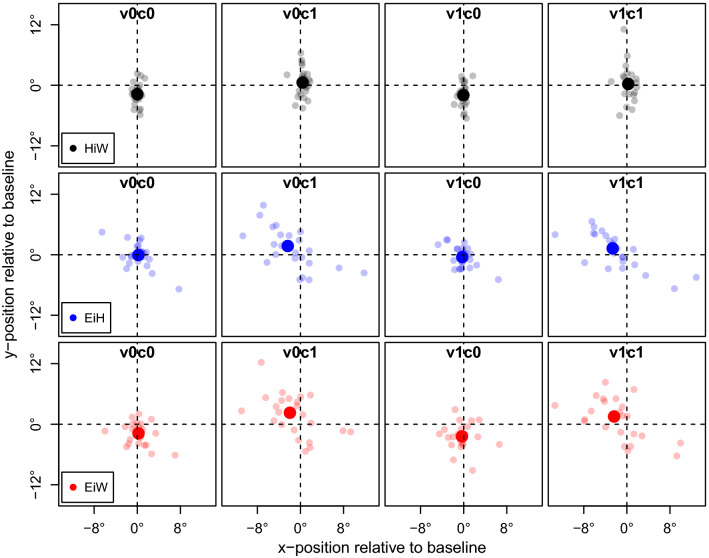
Mean gaze parameters for each block (relative to baseline, i.e., block 1 and 10). For each parameter HiW (black), EiH (blue), EiW (red) baseline-corrected means of the horizontal and vertical orientation for each block type in degree. Each small dot represents one participant, the larger dots represent the mean values across all participants

Mean vertical head orientation (HiW) over entire blocks was significantly tilted more upward during blocks with a *cognitive task* (*F*(1, 23) = 11.36, *p* = 0.003) than without (Fig. 5). As expected the *visual cue* showed no significant longer-term effect (*F*(1, 23) = 1.26, *p* = 0.274) on head orientation and there was also no interaction (*F*(1, 23) = 0.01, *p* = 0.917). In the corresponding absolute means (without baseline correction) in degrees, shown in Table 1, we recognize the same patterns as in Fig. 5.

#### Eye-in-head

For EiH there was neither a significant effect for the *cognitive task* (*F*(1, 23) = 3.74, *p* = 0.066) nor for the *visual cue* (*F*(1, 23) = 2.20, *p* = 0.151). This indicates that, contrary to our expectations, gaze was not significantly elevated in conditions with visual cue (with no interaction: *F*(1, 23) = 0.01, *p* = 0.924), see also Fig. 5, middle row.

#### Eye-in-world

**Fig. 6:**
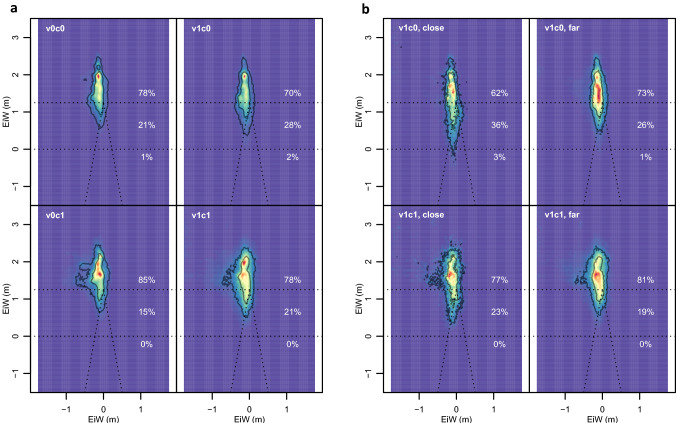
Distribution of gaze orientation by condition. Distribution of gaze (EiW) depending on condition and distance of visual cue. Plotted are absolute coordinates (in meters). Colors show visual density over the entire block from blue (low) to red (high). The upper dashed line represents the horizon, the lower the transition between the treadmill and the screen. **a** Overall gaze distribution, **b** This distinguishes whether the visual cue was visible on the floor projection (“close”) or still on the screen (“far”). The highest view density is slightly above the horizon, for visual cues the view tends to be lower and decreased somewhat more if they get closer. This is perhaps related to tracking of the visual cues in the v1-conditions when the visual cue is close to the participant (supplementary movie S3)

For EiW we see, similar to HiW, a strong effect of the *cognitive task* (*F*(1, 23) = 22.61, *p* < 0.001; Fig. 5, bottom)*.* Further, we replicated the effect of the *visual cue* (*F*(1, 23) = 4.71, *p* = 0.041) on gaze orientation from earlier findings (Kopiske et al. 2021) which in sum reinforced the pattern of the previous parameters (without significant interaction: *F*(1, 23) = 0.03, *p* = 0.870). To investigate how the gaze (EiW) distribution behaves across conditions, we also created gaze maps that visualize the distribution of the gaze data (Fig. 6). Overall, we found gaze pointing straight ahead, mainly aligned around the vertical axis. The focus was slightly above the horizon and the gaze in v1 conditions was slightly lower than for v0 conditions. The gaze then lowers a bit more the closer the visual cue gets (Fig. 6b).

Noticeable is the small gaze shift to the left slightly above the horizon, especially in the c1-conditions. Possibly this is due to irregularities in the virtual sky (clouds were arranged asymmetrically around the vertical midline, see Fig. 1a).

### Adaptation of gaze and gait to motor perturbations

After investigating the patterns of gaze and gait parameters both event-related (slip-locked) and block-wise, we examined short-term and long-term differences in these patterns. How do we adapt our behavior when the same perturbations occur repeatedly?

**Fig. 7:**
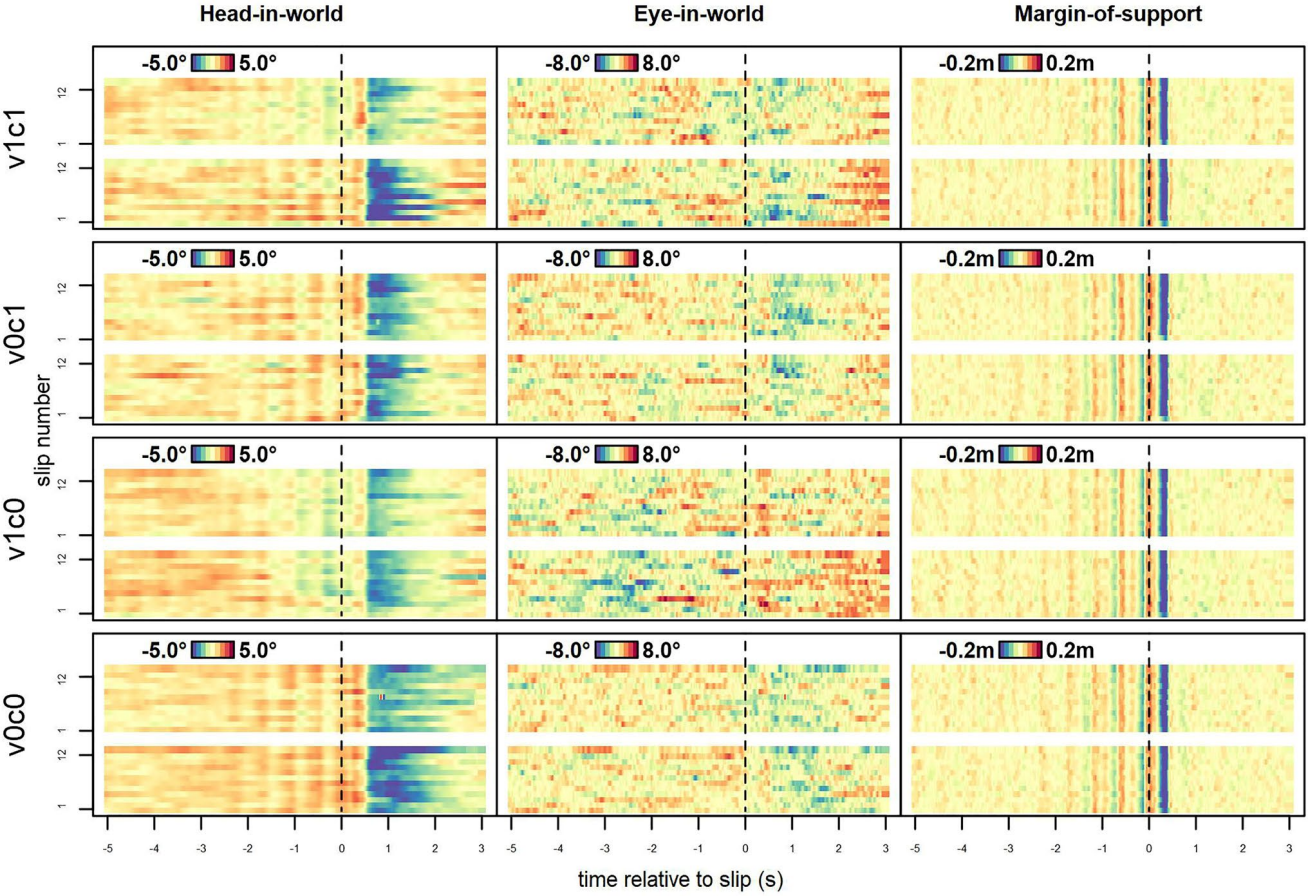
Gaze and gait parameters relative to slips, by slip number. Mean parameters of the first 12 slips for each block, row by row per condition, averaged over all participants. The *x*-axis shows the time relative to the perturbation (dashed vertical lines), the *y*-axis the slip number in each block. The colors represent the baseline-corrected expression of the vertical gaze parameters in degrees as well as the MOS_ap_ in meters which were adjusted to the range of each parameter. The bottom row within each condition shows the first of two blocks of all participants in that condition for each parameter. We found a brief elevated gaze after perturbations (red), with a subsequent lowered gaze (blue), as well as the first slip showing a different pattern to subsequent slips across all conditions. Also seen is a synchronization of steps in the MOS_ap_ as a striped pattern before perturbations. An alternative representation of these data, which delineate the first perturbation event per block more clearly, is shown in the Fig. 9 in Appendix

Thus, we assessed how parameters changed across slips of the same conditions by averaging the responses across all participants for each slip of the same serial position within the block; that is, we averaged the first slip in a block across participants, the second slip and so on up to the twelfth slip in the block (Fig. 7). In general, for most of the slips, we found the pattern that was already seen for the averaged event-related trajectories (Fig. 2). The head orientation HiW showed the characteristic short rise after perturbation, followed by a pronounced downward movement and slower recovery (left columns). It is noticeable that the pattern is less clear for EiW (middle column) than for HiW when combining HiW and EiH, where the gaze lowered not as much as the head orientation and the pattern is more noisy. Looking at gait stability (third column), there was again the slight synchronicity of steps before the slip and the abrupt loss of stability, which recovered very quickly across all conditions.

**Fig. 8:**
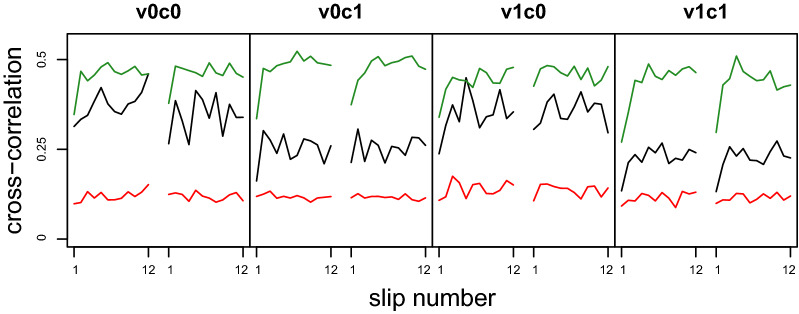
Mean within-participant cross-correlations by slip, condition, and variable. Mean cross-correlations (maximum offset: 0.2 s) of a slip with all other slips of the same participant in the same condition to determine how typical that slip is, separated for MOS_ap_ in green, HiW in black, and EiW in red

Looking closely at the first slip in each condition in Fig. 7 (bottom lines marked with “1” in each panel), this appears to be qualitatively different from the following ones. This would be in line with previous results (Kopiske et al. 2021; Marigold and Patla 2002) that reactions to the first slip in a block tend to be mostly strong, but much more irregular, compared to the subsequent slips. To examine this observation in more detail, we made a comparison between each slip and all other slips in the block to see how typical each slip is (Fig. 8). To this end, we calculated the median cross-correlation between each slip and all other slips of the same participant in the same condition, ensuring that the trajectories were not separated by a whole step (maximum lag of 0.2 s). Across variables and conditions, these medians ranged from *r* = 0.11 for EiW to *r* = 0.49 for MOS_ap_. Within a condition, the cross-correlation of the first slip with all subsequent slips was much lower compared with the cross-correlations for all other slips, seeing a marked jump from the first slip to the second but hardly any increase thereafter (Fig. 8). Comparing c0 and c1-conditions, we see substantially lower median cross-correlation for HiW while participants were performing a cognitive task (median: 0.23 for v1c1 and 0.26 for v0c1, but 0.35 for v1c0 and 0.37 for v0c0). Thus, slip-related head movements did not reach a ‘typical’ pattern to the same degree when cognitive load was higher as for low cognitive load. Cross-correlations between slips of different participants were generally lower (ranging from *r* = 0.08 for EiW to *r* = 0.26 for MOS_ap_) and showed a similar pattern, with little difference between conditions except in HiW, where cross-correlations were lower for c1-conditions (0.16 and 0.18, respectively, compared to 0.2 and 0.25 for the corresponding c0-conditions).

### Summary: cognitive tasks and effector-specific gaze and gait changes

Taken together, we found that both gaze and gait respond to perturbations. Eye movements first showed a clear downward movement due to the perturbations (on the order of 200–300 ms) but afterward a compensatory pattern to the lowered head orientation. This is also reflected in the EiW orientation, although not as strongly. Additionally, head movements were affected by the *cognitive task*, both directly following slips and across entire blocks, unlike eye movements, which in turn responded to *visual cues*, but only directly following a perturbation. Furthermore, an adaptation of the response parameters to the perturbations is observed, which happens quickly and with little carryover between the blocks. Notably, patterns were less similar when participants performed a cognitive task, indicating that variability of the responses to slipping increases when cognitive load is high compared to low-load conditions.

## Discussion

We investigated the effects of a cognitive task on gaze-gait interactions during perturbed naturalistic walking in a virtual environment. A rapid and flexible adaptation of gait due to motor perturbations was replicated, but we see no clear signs of a less stable gait or a cautious gait mode under dual-task conditions. An effect of the cognitive task on gaze orientation was found, as participants looked up more while counting. Further, participants showed no problems completing the cognitive task and this was not affected by the perturbations, whereas perturbation responses were affected by the cognitive task, as it led to less typical slip-patterns developing over slips. Both eye and head movements responded to motor perturbations directly, but in a partially compensatory pattern.

Our findings suggest that cognitive tasks, presumably inducing higher cognitive load, lead to a reduction of slip-adjustments to perturbations—one may say impaired learning—as well as a raised gaze as a strategy to improve task performance through reducing sensory input (Glenberg et al. 1998, p. 651). That said, our data do not allow us to distinguish between participants using fewer visual information in these conditions and them relying on peripheral vision. As our young and healthy participants were sufficiently able to complete the cognitive task, this caused no greater impact on dynamic stability on gait to perturbations. Descriptively, the block-wise difference in MOS_ap_ (Table 1) showed a small increase in gait stability, adopting to a more cautious gait mode, but the statistical test does not offer clear support for this suggestion.

Using a virtual, but naturalistic environment allows high experimental control over perturbations, enabling us to investigate gaze and gait adjustments at different time scales. Again, typical patterns related to perturbations and adjustments to recurring slips can be found, as well as patterns found in previous work in similar environments. We tend to look more where task-relevant information appears (Marigold and Patla 2007) and gait stability is briefly reduced by the perturbations (Kopiske et al. 2021). We confirmed adaptation of slip responses within blocks rather than between blocks, showing rapid but flexible gait adjustments. Notably, time-correlated HiW patterns showed a slight upward shift after the slip followed by a pronounced downward movement with a somewhat slower recovery. The former typically occurred within 200 ms after perturbation and may be reflex based (Nashner 1976). Biomechanical explanations are unlikely, as the strength of immediate gaze responses to the perturbation was only weakly correlated with the strength of gait responses. As expected (Kopiske et al. 2021), visual cues to perturbations were tracked by gaze (especially in the v1c0-condition; i.e., in the absence of a cognitive task) showing a slightly lower gaze prior to perturbations in eye as well as head movements, because they provided reliable information about the perturbations (supplementary movie S3). While head orientation dropped steadily until just before the slip, tracking for eye movements ended slightly earlier. This is not surprising, as difficult terrain is more likely to be fixated at some distance in front of one's feet (Matthis et al. 2018). Gaze (EiW) was also significantly raised over entire blocks while completing a cognitive task. Both are evident in the gaze maps in Fig. 6, which also underline the pattern for tracking visual cues, as gaze was clearly lowered especially when visual cues were near. The gaze maps also show a shift of gaze to the left, likely due to irregularities in the virtual sky (Fig. 1a). Thus, we see some differences, but overall good agreement with the results obtained for slipping without secondary tasks but with otherwise the same methods by Kopiske et al. (2021). One key difference was that here, we found a partially compensatory pattern for eye and head orientation while slipping (see the relatively smaller downward shift in EiW compared to HiW). Gaze was raised rapidly after the brief downward shift following perturbations, but noticeably this is not found in v1c0-conditions, possibly because with visual cues and low cognitive load, participants were less surprised to slip. This downward shift was not seen in our previous study (Kopiske et al. 2021). There are several potential reasons for this, one being that in the present study, we used a newer, lighter, and better-fitting mobile eye tracker, which could have been better suited to measuring such effects. We also found that participants did not tend to look up more (EiH) when visual cues were present.

We also investigated how participants adapted to the repeated perturbations for each condition and also for the repeated occurrence of the same condition. This confirms the special role of the first slip, which has already been pointed out in previous studies (Kopiske et al. 2021; Marigold and Patla 2002). Thus, participants rapidly found an adaptive response to the perturbation for the specific condition but transferred it only minimally, even in v1 blocks where they knew ahead of the first perturbation which exact condition they were in. Interestingly, adjusting gait to perturbations while counting is less pronounced as slips are more dissimilar to each other—especially for HiW. This bears out in lower cross-correlations of slips both within and across participants while performing a cognitive task.

Our cognitive task of counting backward in steps of seven resulted in a raised gaze, mainly through head movements. This is consistent with previous work showing raised gaze as a response to increased cognitive load as a way to “…enhance the efficiency of cognitive processing…” (Glenberg et al. 1998, p. 651) by reducing sensory input that needs to be processed and for visualization of the task. So even if perturbations were visually cued while counting, one could speculate that this could be distracting as well as useful when participants were looking to avoid additional visual input. Despite this, our participants were able to complete the counting task without problems, showing few errors and a relatively steady counting speed (see section “Cognitive dual task”). Note, however, that our participants were all healthy and relatively young—the same task in older or impaired participants might yield a different pattern due to differences in cognitive and motor abilities, as well as a higher cost of falling (Soangra and Lockhart 2017). A comparison between different age groups regarding a displacement of cognitive resources as well as gait difficulty may be a possible target of further investigations, for which our safe and controlled, yet naturalistic, setup is ideally suited. Similarly, it may be worth investigating if the pattern holds in more ecologically valid real-life tasks such as typing a message on a mobile phone (Crowley et al. 2019).

## Conclusion

Induced motor perturbations, visual cue stimuli, and to a lesser extent cognitive tasks showed an influence on gait and gaze parameters in a virtual but naturalistic environment. In particular, counting during perturbed walking led to a raised gaze and a stronger reaction to motor perturbations in head and eye movements, but showed no impact on dynamic gait stability in our young and healthy participants. A partially compensatory movement of the two effectors, eye and head, was shown in response to the perturbations. This response was adjusted quickly and flexibly, with notable differences depending on whether participants were also completing a secondary task, and with only little transfer between identical conditions.

### Electronic supplementary material

Below is the link to the electronic supplementary material.

Supplementary file1 (MP4 10269 KB)

Supplementary file2 (MOV 22597 KB)

Supplementary file3 (MP4 24741 KB)

## Figures and Tables

**Fig. 9:**
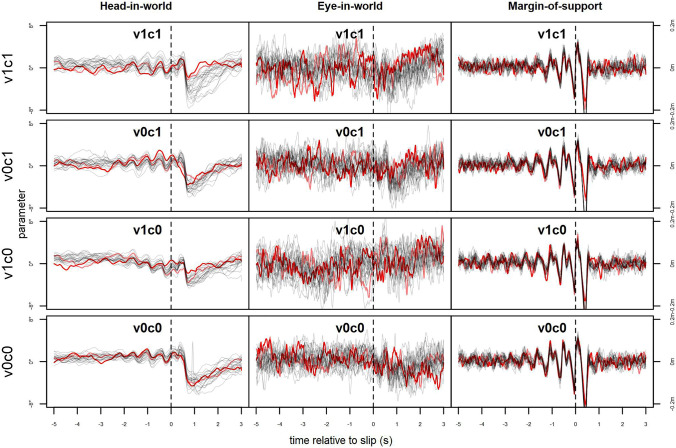
Gaze and gait parameters relative to perturbation, by slip number. Trajectories of the relevant gaze and gait parameters in each condition, by slip number. The red lines indicate the first slip overall (dark red) and the first slip in the second block of the same condition (light red), baseline corrected and averaged across participants. The same data are shown in a different format in Fig. 7. Gray lines show slips 2 through 12 of each of the blocks (resulting in 22 gray lines and two red lines per condition, each representing one slip number). The *x*-axis shows the time relative to the perturbation (dashed vertical lines). The *y*-axis shows the parameter over time relative to the window mean, in degrees for HiW and EiW, in meters for MOS_ap_. Especially in HiW and MOS_ap_, the red lines display a recovery pattern that differed somewhat from other slips, illustrating again the point made in Fig. 8 that most adjustments happened after the first slip in each block. We also again see the synchronization of steps in MOS_ap_ and HiW close to the time of perturbation

## Data Availability

All data and analyses are available at the Open Science Framework: https://osf.io/khn8a/.

## Appendix

See Fig. 9.

## Footnotes

1 The technical latency of 50 ms (see section Environmental setup and materials) between step signal (force > 100 N) and start of acceleration is uncritical, as practically no step (about 1% maximally, but this includes misdetected steps that were only partially on one belt, which typically occur only immediately after perturbations) will take less than 50 ms from crossing 100 N to exerting the maximum force (median time is 184 ms for unperturbed steps).
